# Genetic (co)variance of rainbow trout (*Oncorhynchus mykiss*) body weight and its uniformity across production environments

**DOI:** 10.1186/s12711-015-0122-8

**Published:** 2015-05-19

**Authors:** Panya Sae-Lim, Antti Kause, Matti Janhunen, Harri Vehviläinen, Heikki Koskinen, Bjarne Gjerde, Marie Lillehammer, Han A Mulder

**Affiliations:** Nofima Ås, Osloveien 1, P.O. Box 210, NO-1431 Ås Norway; Natural Resources Institute Finland (LUKE), Biometrical Genetics, FI-31600 Jokioinen, Finland; Natural Resources Institute Finland (LUKE), Aquaculture Unit, FI-72210 Tervo, Finland; Animal Breeding and Genomics Centre, Wageningen University, P.O. Box 338, 6700 AH Wageningen, the Netherlands

## Abstract

**Background:**

When rainbow trout from a single breeding program are introduced into various production environments, genotype-by-environment (GxE) interaction may occur. Although growth and its uniformity are two of the most important traits for trout producers worldwide, GxE interaction on uniformity of growth has not been studied. Our objectives were to quantify the genetic variance in body weight (BW) and its uniformity and the genetic correlation (*r*_g_) between these traits, and to investigate the degree of GxE interaction on uniformity of BW in breeding (BE) and production (PE) environments using double hierarchical generalized linear models. Log-transformed data were also used to investigate whether the genetic variance in uniformity of BW, GxE interaction on uniformity of BW, and *r*_g_ between BW and its uniformity were influenced by a scale effect.

**Results:**

Although heritability estimates for uniformity of BW were low and of similar magnitude in BE (0.014) and PE (0.012), the corresponding coefficients of genetic variation reached 19 and 21%, which indicated a high potential for response to selection. The genetic re-ranking for uniformity of BW (*r*_g_ = 0.56) between BE and PE was moderate but greater after log-transformation, as expressed by the low *r*_g_ (-0.08) between uniformity in BE and PE, which indicated independent genetic rankings for uniformity in the two environments when the scale effect was accounted for. The *r*_g_ between BW and its uniformity were 0.30 for BE and 0.79 for PE but with log-transformed BW, these values switched to -0.83 and -0.62, respectively.

**Conclusions:**

Genetic variance exists for uniformity of BW in both environments but its low heritability implies that a large number of relatives are needed to reach even moderate accuracy of selection. GxE interaction on uniformity is present for both environments and sib-testing in PE is recommended when the aim is to improve uniformity across environments. Positive and negative *r*_g_ between BW and its uniformity estimated with original and log-transformed BW data, respectively, indicate that increased BW is genetically associated with increased variance in BW but with a decrease in the coefficient of variation. Thus, the scale effect substantially influences the genetic parameters of uniformity, especially the sign and magnitude of its *r*_g_.

## Background

Uniformity of traits depends on an individual’s sensitivity to perturbations in its internal environment and of unknown local micro-environmental factors [1-3]. Additive genetic variance in uniformity is defined as the genetic heterogeneity of the residual variance for a trait [4-6]. Accordingly, less sensitive genotypes have offspring that are more uniform and show a smaller within-family residual variance. In animal production, uniformity is often a desired character because: (1) it indicates phenotypic robustness and (2) it aids in producing homogeneous animal stocks and uniform food products. Generally, aquatic animals exhibit considerable heterogeneity in growth performance. Large variation in body weight (BW) among individuals reduces fish welfare and decreases the sustainability of the aquaculture industry [7]. A common practice to reduce heterogeneity in growth performance is regular grading during rearing, where heterogeneous fish schools are size-sorted into more homogeneous groups that are harvested at different times. However, these procedures reduce overall profit at the farm scale because labor and farming costs increase and the price of small-sized fish is lower. Another solution would be to select animals for uniformity of growth performance, provided that some genetic variance exists for this trait.

Rainbow trout, *Oncorhynchus mykiss* (Walbaum 1792), is an economically important aquaculture species, and breeding programs distribute improved material across continents [8]. Producers worldwide of rainbow trout regard growth performance and its uniformity as two of the most important traits to be improved by selective breeding [9]. When rainbow trout from a single breeding program are introduced into various production environments, a genotype by environment (GxE) interaction on growth performance and its uniformity may occur, which hampers genetic improvement of these traits across multiple environments. However, to our knowledge, GxE interaction on uniformity of growth performance in rainbow trout has not been studied.

In Finland, rainbow trout is farmed in sea and in inland freshwater environments. The nucleus of the Finnish national breeding program is located inland, where breeding candidates are kept in fresh water, while their sibs are performance-tested in the Baltic Sea, where most large-scale commercial production takes place. To reduce the risk of disease infections, sea-tested individuals and their eggs or milt are not transported from the sea test station back to the nucleus. Genetic parameters for uniformity of BW in rainbow trout have been studied only in the nucleus environment [10], using the additive model described by Mulder et al. [11]. The statistical methods applied for genetic analysis of uniformity of BW have, however, evolved rapidly in recent years. Rönnegård et al. [12] introduced a double hierarchical generalized linear model (DHGLM), which has the advantage of accounting for the non-normal distribution of squared residuals. Therefore, in this study, genetic parameters for uniformity of BW that were previously estimated in the nucleus environment were confirmed by using multivariate DHGLM, and uniformity of BW was studied on fish from both the freshwater nucleus and sea test stations. For many morphological traits, a positive correlation between mean and variance is expected, i.e. the variance increases with the mean, which is referred to as a scale effect [13]. When trait variation is scaled by the mean trait value, e.g. by log-transforming the data, the resulting log-transformed variance quantifies the variation that does not depend on the scale effect [13,14].

Thus, the aims of this study were: (1) to quantify the genetic variance of harvest BW and its uniformity and the genetic correlation between these traits for sibs reared in the freshwater breeding nucleus and in the seawater production environment, and (2) to investigate the degree of GxE interaction on uniformity of BW for these two environments. Finally, we also log-transformed the data to investigate whether genetic variance in uniformity of BW, GxE interaction on uniformity of BW, and the genetic correlation between BW and its uniformity were influenced by the scale effect, micro-environmental sensitivity, or both.

## Methods

### Data

The data used in this study originated from the Finnish national breeding program [15,16]. All procedures that involved animals were approved by the animal care committee of the Finnish Game and Fisheries Research Institute (FGFRI). The breeding nucleus (defined as the breeding environment or BE) was located on the FGFRI fish farm located in Tervo (Central Finland). Siblings of the breeding candidates were tested in commercial sea cages (defined as the production environment or PE) located in the Baltic Sea. Phenotypic data comprised 53 638 records on BW at tagging from four year classes and belonged to two subpopulations, i.e., 1996/1999 and 1997/2000, which were measured for both environments. For each year class, the number of sires and dams and the number of recorded offspring are in Table 1. Both subpopulations were established from the parents of year class 1993. Sires and dams were mated using either paternal nested or partial factorial mating designs. Each year class consisted of 94 to 197 full-sib families established from the mating of 37 to 95 sires with 79 to 129 dams. After hatching, fingerlings from the same full-sib family were maintained in one or more family tanks until they reached a body size suitable for individual passive integrated transponder (PIT) tagging (i.e. at a mean BW of approximately 50 g). During tagging, full-sibs from each family were randomly sampled and divided into two or three batches that were reared either in BE or PE. When the fish were 2 years old, they were individually weighed, denoted by BW_BE_ in g for BE and BW_PE_ for PE. For BE, sex and sexual maturity status were recorded based on external sex characters, whereas for PE, since fish were slaughtered, sex and maturity were identified based on the morphology of the gonads.

**Table 1:** **Population structure of rainbow trout**

	**Subpopulation I**		**Subpopulation II**	
	**1996**	**1999**	**1997**	**2000**
*Number of parents and families*				
Sires, dams	57, 129	37, 94	65, 79	95, 121
Sires per dam, mean (range)	1.00 (1-1)	1.00 (1-1)	2.41 (1-3)	1.63 (1-3)
Dams per sire, mean (range)	2.26 (1-4)	2.54 (1-4)	2.93 (1-5)	2.06 (1-5)
Full-sib families, family tanks	129, 129	94, 135	191, 259	197, 197
*Number of fish with records*				
Freshwater nucleus station	4994	3084	8099	5998
Fish per full-sib family	38.7	32.8	42.4	30.4
Seawater station	2573	2442	8351	7499
Fish per full-sib family	19.9	26.0	43.7	38.1

In total, 22 175 and 20 865 individual records were available for BW_BE_ and BW_PE_, respectively (Table 1). Average BW_BE_ and BW_PE_ (standard deviation, SD) estimated from the raw data were equal to 1094 (364) and 1050 (335) g, respectively.

### Statistical analysis

We used DHGLM for statistical analysis [12,14,17], which uses a log-link function to account for the non-normal distribution of squared residuals. The models were run for two transformations for the data. First, observed BW were standardized to a mean of 0 and variance of 1 to rescale the original data, which facilitates convergence. Second, observed BW were log-transformed to account for the scale effect. Log-transformation is one way to reduce dependency of variance on mean, since the log-variance represents a parameter that is similar to the coefficient of variation [13]. Both standardized and log-transformed data were modelled using the following multivariate sire-dam DHGLM in ASReml [18,19]:

$$ \begin{array}{l}\left[\begin{array}{c}\hfill {\mathbf{y}}_{E_1}\hfill \\ {}\hfill {\boldsymbol{\Psi}}_{E_1}\hfill \\ {}\hfill {\mathbf{y}}_{E_2}\hfill \\ {}\hfill {\boldsymbol{\Psi}}_{E_2}\hfill \end{array}\right]=\\ {}\left[\begin{array}{c}\hfill {\mathbf{X}}_{E_1}\hfill \\ {}\hfill \hfill \\ {}\hfill sym\hfill \\ {}\hfill \hfill \end{array}\begin{array}{c}\hfill \mathbf{0}\hfill \\ {}\hfill {\mathbf{X}}_{v,{E}_1}\hfill \\ {}\hfill \hfill \\ {}\hfill \hfill \end{array}\begin{array}{c}\hfill \mathbf{0}\hfill \\ {}\hfill \mathbf{0}\hfill \\ {}\hfill {\mathbf{X}}_{E_2}\hfill \\ {}\hfill \hfill \end{array}\begin{array}{c}\hfill \mathbf{0}\hfill \\ {}\hfill \mathbf{0}\hfill \\ {}\hfill \mathbf{0}\hfill \\ {}\hfill {\mathbf{X}}_{v,{E}_2}\hfill \end{array}\right]\left[\begin{array}{c}\hfill {\mathbf{b}}_{E_1}\hfill \\ {}\hfill {\mathbf{b}}_{v,{E}_1}\hfill \\ {}\hfill {\mathbf{b}}_{E_2}\hfill \\ {}\hfill {\mathbf{b}}_{v,{E}_2}\hfill \end{array}\right]+\\ {}\left[\begin{array}{cccc}\hfill {\left({\mathbf{Z}}_s+{\mathbf{Z}}_d\right)}_{E_1}\hfill & \hfill \mathbf{0}\hfill & \hfill \mathbf{0}\hfill & \hfill \mathbf{0}\hfill \\ {}\hfill \hfill & \hfill {\left({\mathbf{Z}}_s+{\mathbf{Z}}_d\right)}_{v,{E}_1}\hfill & \hfill \mathbf{0}\hfill & \hfill \mathbf{0}\hfill \\ {}\hfill sym\hfill & \hfill \hfill & \hfill {\left({\mathbf{Z}}_s+{\mathbf{Z}}_d\right)}_{E_2}\hfill & \hfill \mathbf{0}\hfill \\ {}\hfill \hfill & \hfill \hfill & \hfill \hfill & \hfill {\left({\mathbf{Z}}_s+{\mathbf{Z}}_d\right)}_{v,{E}_2}\hfill \end{array}\right]\left[\begin{array}{c}\hfill {\mathbf{u}}_{E_1}\hfill \\ {}\hfill {\mathbf{u}}_{v,{E}_1}\hfill \\ {}\hfill {\mathbf{u}}_{E_2}\hfill \\ {}\hfill {\mathbf{u}}_{v,E2}\hfill \end{array}\right]+\\ {}\left[\begin{array}{cccc}\hfill {\mathbf{Q}}_{E_1}\hfill & \hfill \mathbf{0}\hfill & \hfill \mathbf{0}\hfill & \hfill \mathbf{0}\hfill \\ {}\hfill \hfill & \hfill {\mathbf{Q}}_{v,{E}_1}\hfill & \hfill \mathbf{0}\hfill & \hfill \mathbf{0}\hfill \\ {}\hfill sym\hfill & \hfill \hfill & \hfill {\mathbf{Q}}_{E_2}\hfill & \hfill \mathbf{0}\hfill \\ {}\hfill \hfill & \hfill \hfill & \hfill \hfill & \hfill {\mathbf{Q}}_{v,{E}_2}\hfill \end{array}\right]\left[\begin{array}{c}\hfill {\mathbf{c}}_{E_1}\hfill \\ {}\hfill {\mathbf{c}}_{v,{E}_1}\hfill \\ {}\hfill {\mathbf{c}}_{E_2}\hfill \\ {}\hfill {\mathbf{c}}_{v,{E}_2}\hfill \end{array}\right]+\left[\begin{array}{c}\hfill {\mathbf{e}}_{E_1}\hfill \\ {}\hfill {\mathbf{e}}_{v,{E}_1}\hfill \\ {}\hfill {\mathbf{e}}_{E_1}\hfill \\ {}\hfill {\mathbf{e}}_{\mathrm{v},{E}_2}\hfill \end{array}\right],\end{array} $$

where **y**_***Ej***_ is the vector of BW phenotypes or *y*_ij_ of the *i*^th^ individual measured in the *j*^th^ environment (*E*_j_ *=* BE or PE). **ψ** is the vector of the residual variances ${\phi}_{ij}=\left(\frac{{\widehat{e}}_{ij}^2}{1-{h}_{ij}}\right)$, which was linearized using a Taylor series approximation in ASReml, where ${\widehat{e}}_{ij}^2$ is the squared residual estimate of BW_,_ and *h*_ij_ is the diagonal element in the hat-matrix (predicted value matrix) of **y**_Ej_ corresponding to *y*_ij_. **X**(**X**_*v*_) is the incidence matrix of fixed interaction effects of year class, site (freshwater and seawater), sex (male, female, or unknown), and maturity (maturity at 2 or 3 years of age, or unknown). Within-family additive genetic variance can be influenced by parental inbreeding [3], but in our data, parental average inbreeding coefficients were equal to 0 [15], and thus this coefficient was excluded from the model. **b**(**b**_*v*_) is the solution vector of fixed interaction effects. **Z**_S_ and **Z**_*d*_ are the incidence matrices of random sire (*s*) and dam (*d*) effects, and **u**(**u**_*v*_) is the vector of additive genetic effects of sires and dams on BW (uniformity) and was assumed to have the following distribution:

$$ \left[\begin{array}{c}\hfill {\mathbf{u}}_{E_1}\hfill \\ {}\hfill {\mathbf{u}}_{v,{E}_1}\hfill \\ {}\hfill {\mathbf{u}}_{E_2}\hfill \\ {}\hfill {\mathbf{u}}_{v,{E}_2}\hfill \end{array}\right]\sim \mathrm{N}\left(\mathbf{0},\frac{1}{4}\mathbf{G}\otimes \mathbf{A}\right), $$

where **G** is 4×4 sire-dam (co)variance matrix, and **A** is the numerator relationship matrix. **Q**(**Q**_*v*_) is the incidence matrix of random effects common to full-sibs (family tank prior to communal rearing and non-additive genetic effects) other than additive genetic effects, and **c**(**c**_*v*_) is the vector of solutions for the effect common to full-sibs, $$ \left[\begin{array}{c}\hfill {\mathbf{c}}_{E_1}\hfill \\ {}\hfill {\mathbf{c}}_{v,{E}_1}\hfill \\ {}\hfill {\mathbf{c}}_{E_2}\hfill \\ {}\hfill {\mathbf{c}}_{v,{E}_2}\hfill \end{array}\right]\sim \mathrm{N}\left(\mathbf{0},\mathbf{C}\otimes \mathbf{I}\right) $$, where **C** is 4×4 (co)variance matrix of the effect common to full-sibs and **I** is an identity matrix. The residual of **y** (**e**) and **ψ**(**e**_*v*_) were assumed independently normally distributed:

$$ \left[\begin{array}{c}\hfill {\boldsymbol{e}}_{E_1}\hfill \\ {}\hfill {\boldsymbol{e}}_{v,{E}_1}\hfill \\ {}\hfill {\boldsymbol{e}}_{E_2}\hfill \\ {}\hfill {\boldsymbol{e}}_{v,{E}_2}\hfill \end{array}\right]\sim \mathrm{N}\left(\begin{array}{c}\hfill \mathbf{0}\hfill \\ {}\hfill \mathbf{0}\hfill \\ {}\hfill \mathbf{0}\hfill \\ {}\hfill \mathbf{0}\hfill \end{array},\left[\begin{array}{cccc}\hfill {\mathbf{W}}_{E_1}^{-1}{\sigma}_{\in}^2\hfill & \hfill \mathbf{0}\hfill & \hfill \mathbf{0}\hfill & \hfill \mathbf{0}\hfill \\ {}\hfill \hfill & \hfill {\mathbf{W}}_{v,{E}_1}^{-1}{\sigma}_{\in_v}^2\hfill & \hfill \mathbf{0}\hfill & \hfill \mathbf{0}\hfill \\ {}\hfill sym\hfill & \hfill \hfill & \hfill {\mathbf{W}}_{E_2}^{-1}{\sigma}_{\in}^2\hfill & \hfill \mathbf{0}\hfill \\ {}\hfill \hfill & \hfill \hfill & \hfill \hfill & \hfill {\mathbf{W}}_{v,{E}_2}^{-1}{\sigma}_{\in_v}^2\hfill \end{array}\right]\right), $$

where ${\mathbf{W}}_{Ej}=\mathrm{diag}\left({\widehat{\boldsymbol{\uppsi}}}_{Ej}^{-1}\right)$, ${\mathbf{W}}_{v,Ej}=\mathrm{diag}\left(\frac{1-{\mathbf{h}}_j}{2}\right)$, and ${\sigma}_{\in}^2\left({\sigma}_{\in_v}^2\right)$ is a scaling variance that was expected to be 1, since **W** contains the reciprocal of the individual residual variances. The multivariate sire-dam DHGLM was fitted iteratively, with updating of **ψ** and the diagonal elements of **W**_E*j*_ and **W**_*v,Ej*_ until the log-likelihood converged [19].

### Calculation of estimates of genetic parameters

The estimated additive genetic sire-dam variance component $\left({\sigma}_{u_v, \exp}^2\right)$ for uniformity of BW was on the exponential scale (exp) and was consequently converted to an additive scale $\left({\sigma}_{u_v}^2\right)$ using the equations derived by Mulder et al. [5]. The estimated variance component for the effect common to full-sibs $\left({\sigma}_{c{}_v, \exp}^2\right)$ for uniformity of BW was converted to an additive scale $\left({\sigma}_{c_v}^2\right)$ using the equation derived in the Appendix.

In the sire-dam model, the estimated variance component for sires was set equal to the variance component for dams and equal to one quarter of the additive genetic variance $\left({\sigma}_s^2={\sigma}_d^2={\sigma}_u^2=\frac{1}{4}{\sigma}_a^2\right)$, and the estimated variance component for uniformity of BW $\left({\sigma}_{u_v}^2\right)$ was equal to one quarter of the genetic variance for uniformity of BW. Therefore, additive genetic variance components estimated for BW $\left({\sigma}_a^2\right)$ and for uniformity of BW $\left({\sigma}_{a_v}^2\right)$ were equal to $4{\sigma}_u^2$ and $4{\sigma}_{u_v}^2$, respectively. Phenotypic variance $\left({\sigma}_p^2\right)$ of BW was equal to $2{\sigma}_u^2+{\sigma}_c^2+{\sigma}_e^2$, where ${\sigma}_c^2$ is the variance component for the effect common to full-sibs and ${\sigma}_e^2$ is the residual variance of BW, which in a sire-dam model includes one half of the additive genetic variance plus the random environmental variance. Therefore, ${\sigma}_u^2$ was multiplied by 2 to calculate ${\sigma}_p^2$. Heritability for BW (*h*^2^) was calculated as ${\sigma}_a^2/{\sigma}_p^2$ and for uniformity of BW $\left({h}_v^2\right)$ as $\frac{\sigma_{a_v}^2}{2{\sigma}_p^4+3\left({\sigma}_{a_v}^2+{\sigma}_{c_v}^2\right)}$ (see [20] and Appendix). Similarly, the common environmental effect for BW (*c*^2^) was calculated as ${\sigma}_c^2/{\sigma}_p^2$ and for uniformity of BW $\left({c}_v^2\right)$ as $\frac{\sigma_{c_v}^2}{2{\sigma}_p^4+3\left({\sigma}_{a_v}^2+{\sigma}_{c_v}^2\right)}$ (see Appendix).

The genetic coefficient of variation for uniformity of BW ($C{V}_{a_v}$) was calculated as ${\sigma}_{a_v}/{\sigma}_E^2$, where ${\sigma}_E^2={\sigma}_e^2-2{\sigma}_u^2$. Three genetic correlations (*r*_g_) were calculated based on the additive genetic covariance divided by the product of the two corresponding additive genetic standard deviations: (1) between BW and its uniformity within an environment, (2) for one trait (BW or its uniformity) in BE versus PE, i.e. to quantify genetic re-ranking between environments, and (3) between BW in one environment and its uniformity in the other environment.

Approximate standard errors of variance component estimates were calculated with ASReml following Fisher et al. [21]. Approximated standard errors of ${h}_v^2$ and ${c}_v^2$ are not available in ASReml and to our knowledge have not been derived.

### Comparison with an additive model

We also used an additive model that refers to the “iterative bivariate model” described in Mulder et al. [11], to analyze part of the data for BW and its uniformity using the log-squared residuals, as described in [10,11]. In that analysis, we included only the data from BE and we report only the genetic correlation between BW and its uniformity. Our aim was to verify whether or not the genetic correlation estimated by the additive model was consistent with that estimated by the DHGLM when the scale effect on uniformity of BW was accounted for by log-transformation of BW.

## Results

### Genetic variation of BW and its uniformity

Using standardized data, additive genetic variances for BW were similar for BE and PE (0.140 to 0.143). Heritability estimates for BW (*h*^2^) were also similar and moderate for BE (0.258) and PE (0.221) (Table 2). Heritability estimates for uniformity of BW $\left({h}_v^2\right)$ were low and of similar magnitude for BE (0.011) and PE (0.010). Using log-transformed data, additive genetic variances in BW were proportionally lower for both environments (0.013 to 0.015) than with standardized data. All estimates of genetic variance for uniformity of BW were greater than their standard errors (SE). Using log-transformed data, estimates of *h*^2^ for BW in BE and PE were slightly lower (0.12 to 0.17) than with standardized data, and the estimate of $\left({h}_v^2\right)$ for uniformity of BW in BE (0.024) was slightly higher than with standardized data, whereas $\left({h}_v^2\right)$ in PE (0.010) was similar to that obtained with standardized data.

**Table 2:** **Estimates of variance components and genetic parameters of body weight at harvest and its uniformity measured under breeding and production environments, with or without log-transformation of the data**

**Trait/Parameter**	**Environment**			
	**Standardized**		**Log-transformed**	
	**Breeding**	**Production**	**Breeding**	**Production**
Body weight				
${\sigma}_p^2$	0.543	0.646	0.089	0.109
${\sigma}_a^2$	0.140	0.143	0.015	0.013
${\sigma}_c^2$	0.019	0.024	0.003	0.003
*h* ^2^ (SE)	0.258 (0.031)	0.221 (0.028)	0.170 (0.023)	0.120 (0.017)
*c* ^2^ (SE)	0.036 (0.007)	0.038 (0.006)	0.031 (0.005)	0.026 (0.004)
Uniformity of body weight				
${\sigma}_{a_v, exp}^2$ (SE)	0.0433 (0.014)	0.0354 (0.012)	0.0814 (0.030)	0.0289 (0.020)
${\sigma}_{a_v}^2$	0.0068	0.0085	0.0004	0.0003
${\sigma}_{c_v}^2$	0.0032	0.0031	0.0004	0.0005
$C{V}_{a_v}$	0.211	0.190	0.296	0.174
${h}_v^2$	0.011	0.010	0.024	0.010
${c}_v^2$	0.005	0.004	0.021	0.019

${\sigma}_p^2$ = phenotypic variance $\left(2{\sigma}_u^2+{\sigma}_c^2+{\sigma}_e^2\right)$, where ${\sigma}_e^2$ is the residual variance for body weight; ${\sigma}_a^2$ and ${\sigma}_{a_v}^2$ = additive genetic variance for body weight and its uniformity, respectively; ${\sigma}_c^2$ = common environmental variance; $C{V}_{a_v}$ = coefficient of additive genetic variance for uniformity $\left({\sigma}_{a_v}/{\sigma}_E^2\right)$, where ${\sigma}_E^2={\sigma}_e^2-2\ast {\sigma}_u^2$; *h* ^2^ = heritability for body weight; *c* ^2^ = common environmental effect due to full-sib tanks; ${h}_v^2$ = heritability for uniformity; ${c}_v^2$ = same as *c* ^2^ but for uniformity of body weight.

While estimates of $\left({h}_v^2\right)$ were low, genetic coefficients of variation $C{V}_{a_v}$ for uniformity of BW estimated with standardized data were equal to 21.1% for BE and 19.0% for PE, which indicated a high genetic potential for response to selection relative to the mean. The $C{V}_{a_v}$ for uniformity of BW estimated with log-transformed data was equal to 29.6% and 17.4% for BE and PE, respectively, which supports the existence of genetic differences for uniformity of BW beyond the scale effect.

Estimates of common environmental effects on BW, *c*^2^, ranged from to 0.02 to 0.03 for both environments, which suggests that a small amount of phenotypic variation was due to separate full-sib tanks before communal rearing and to non-additive genetic effects. The estimate of ${c}_v^2$ for uniformity of BW ranged from 0.004 to 0.005 when estimated based on standardized data and from 0.019 to 0.021 when estimated with log-transformed data.

### Genotype by environment interaction

Using standardized data, the estimate of *r*_g_ of BW between BE and PE was equal to 0.70 ± 0.06, which means that a low degree of re-ranking occurred between environments (Table 3), while slightly greater re-ranking (*r*_g_ = 0.56 ± 0.20) was found for uniformity of BW between BE and PE. Using log-transformed data, the estimate of *r*_g_ of BW between BE and PE was equal to 0.66 ± 0.06, which was similar to that obtained with standardized data. In contrast, the estimate of *r*_g_ of uniformity of BW between BE and PE was close to 0 (-0.08 ± 0.33), which indicates that, after accounting for the scale effect, the ranking of breeding values for uniformity of BW between BE and PE was independent.

**Table 3:** **Estimates of the genetic correlation (** *r*_g_ **) and standard errors between body weight at harvest (BW) and its uniformity, within and between environments and with or without log-transformation of the data**

**Trait**	***r***_**g**_ **(SE)**	
	**Standardized**	**Log-transformed**
***Within environment***		
BW_BE_ – uniformity_BE_	0.30 (0.15)	−0.83 (0.10)
BW_PE_ – uniformity_PE_	0.79 (0.13)	−0.62 (0.21)
***GxE interaction***		
BW_BE_ – BW_PE_	0.70 (0.06)	0.66 (0.06)
uniformity_BE_ – uniformity_PE_	0.56 (0.20)	−0.08 (0.33)
***Different trait - different environment***		
BW_BE_ – uniformity_PE_	0.49 (0.14)	−0.42 (0.25)
BW_PE_ – uniformity_BE_	0.46 (0.15)	−0.31 (0.16)

BE = breeding environment; PE = production environment.

### Genetic correlation between BW and its uniformity

With standardized data, the estimate of *r*_g_ between BW and uniformity of BW was lower in BE (0.30) than in PE (0.79). However, with log-transformed data, the estimates of *r*_g_ between BW and uniformity of BW in the two environments were similar but negative (-0.83 in BE and -0.62 in PE). For transformed data, the magnitude of these estimates was greater than the estimates of *r*_g_ between BW_BE_ and its log-squared residuals from the additive model (-0.40 ± 0.05).

As for genetic correlations within one environment, estimates of *r*_g_ between traits measured in different environments depended on whether standardized or log-transformed data were used (Table 3). With standardized data, the estimates of *r*_g_ between BW_BE_ and uniformity_PE_ (0.49) and between BW_PE_ and uniformity_BE_ (0.46) were similar. Conversely, *r*_g_ estimated with log-transformed data was negative but of similar magnitude between BW_BE_ and uniformity_PE_ (-0.42) and between BW_PE_ and uniformity_BE_ (-0.31).

## Discussion

### Genetic variation for uniformity of body weight

Heritability for uniformity of BW estimated with standardized data was low for both environments (<0.02). After log-transformation, estimates of *h*^2^ and *c*^2^ for BW were lower in both environments, whereas estimates of ${h}_v^2$ and ${c}_v^2$ for uniformity of BW were higher in BE but lower in PE. The estimates of ${h}_v^2$ for uniformity of BW obtained in this study are in line with those previously reported for rainbow trout $\left({h}_v^2=0.024\right)$ [10] and for terrestrial animals such as snail [22], broiler chickens [11,23,24], mice [25], and pigs [26] (${\overline{h}}_v^2=0.028$: min = 0.006 and max = 0.047; reviewed by Hill and Mulder [6]). These ${h}_v^2$ estimates are low partly because the heritability is defined at the level of a single observation and estimating a breeding value for variance based on a single observation will be inaccurate. In addition, uniformity of BW can be affected by multiple environmental factors that reduce heritability estimates [27]. In fact, finding low heritabilities with high *CV*_a_ seems to be a general observation for traits that are closely related to fitness, such as fecundity and age at sexual maturity [27].

Although we found low estimates of ${h}_v^2$ for uniformity of BW, its $C{V}_{a_v}$ was high in both environments (21.1% in BE and 19.0% in PE), which indicates a high potential for genetic gain in response to selection for increased uniformity relative to the mean [5,27,28]. The $C{V}_{a_v}$ estimated in this study are in the lower range of those reported previously for uniformity of BW in rainbow trout (0.374) [10], Atlantic salmon (0.417) [14], and terrestrial animals (${\overline{CV}}_{a_v}$ = 40.6%; min = 30.0% and max = 58.0%) [6,11,22-26].

The $C{V}_{a_v}$ estimated with standardized BW data can be explained by both the scale effect and additive genetic effects of micro-environmental sensitivity. As expected, when the scale effect was accounted for by log-transformation of BW, the additive genetic variance of uniformity of BW decreased. Yet, after log-transformation, $C{V}_{a_v}$ estimates remained high, which indicates that genetic variation for uniformity of BW is scale independent. Previous studies have reported a similar phenomenon by comparing transformed and untransformed data for BW in Atlantic salmon [14] and for litter size in rabbits and pigs [29]. Studies on uniformity commonly use transformed data to reduce non-normality of the data. For example, squared residuals were log-transformed in the additive model applied in [10,11], and a Box-Cox transformation was used in the Bayesian approach of [10,23,28,29]. It is likely that such transformations also removed part of the scale effect from the data, which influences the genetic parameters of uniformity, as evidenced by the current and previous studies [14,29].

The definition of uniformity before and after log-transformation is not the same. From a biological point of view, uniformity of log-transformed BW may be more relevant because the scale effect is accounted for and thus the actual genetic variation for environmental canalization can be quantified. However, for a fish farmer, uniformity at the observed scale may be more relevant because it corresponds to the real range of fish sizes that are processed by the industry. Variance at the observed scale can be selected for directly, whereas log-transformed variance can be controlled either by direct selection, or by selecting on an index with appropriate weights on mean BW and observed variance.

In the sire-dam mixed DHGLM that we applied, the squared residuals that were used as phenotypic observations of uniformity included both the Mendelian sampling term and the true residual that remained unexplained by the systematic fixed effects, the random additive genetic effects, and the non-genetic random effects. The animal mixed DHGLM, assumes that the residual is free of Mendelian sampling variance [14]. However, applying this model may create biased genetic parameters for uniformity [30] and thus requires that repeated records from the same individuals are used to minimise the bias of genetic parameters [12].

A reduction in phenotypic variation of animal traits is beneficial for animal production. However, selection for uniformity in livestock is in its initial phase of development. In rabbits, selection for uniformity of birth weight was implemented successfully and resulted in improved survival rate of baby rabbits without reducing mean birth weight [31]. To our knowledge, selection for uniformity has not been implemented yet in fish breeding. It has been stated that selection for uniformity is not relevant when the profit function based on mean values of a trait is linear [28], which is the case for growth performance or body weight. However, it is arguable that uniformity *per se* has both an economic and a non-economic value [32]. In aquaculture, a more uniform growth pattern reduces the mortality of smaller fish [33]. During the grow-out period, the size of feed pellets is increased as the mean BW of a fish school increases. Thus, a uniform growth pattern allows most fish to adapt to changes in pellet size. Uniformity of growth may also partially reduce negative social interactions between fish and thus the development of behavioural dominance hierarchies [34,35], which further improves fish welfare. Moreover, it has been suggested that uniformity of growth performance reduces the need for size-grading and thus, improves the efficiency of fish production [36]. Therefore, while the economic value of uniformity remains to be calculated, simultaneous selection for BW and its uniformity is expected to yield direct and indirect profitable prospects. Direct selection for uniformity of a trait should be carried out if this trait is economically important and can be recorded at an acceptable cost.

The methods investigated in this study can also be extended to other trait types. For example, reducing the variation in carcass quality traits that have intermediate optimum values such as fillet color, fillet lipid content and body shape, has economic value, i.e. fillet color and fillet lipid content should not be too low or too high, and body shape should not be too thin or too round.

Considering the breeders equation Δ*G* = *ir*_IH_*σ*_*a*_ [3], response to selection is determined by three factors: selection intensity (*i*), accuracy of selection (*r*_IH_), and the genetic standard deviation (*σ*_*a*_) or *CV*_*a*_ when Δ*G* is relative to a trait mean. We found that the $C{V}_{a_v}$ for uniformity of BW was high, which indicates that substantial genetic response to selection relative to the mean can be expected. To minimize sampling variance of the estimates of ${\sigma}_{a_v}$, phenotypes on a large number of relatives are required. Based on the equation in Hill and Mulder [6], the optimal family size to estimate variance components for uniformity of BW is 85 for full-sibs when using average *h*^2^ estimates of 0.145 for log-transformed BW and a $C{V}_{a_v}$ of 23.5% for log-transformed uniformity of BW (Table 2). In livestock, e.g., for dairy cattle, information is mainly available from half-sibs, for which the optimal design is 190 half-sibs for the same input parameters.

With respect to the *r*_IH_, accuracies of sib selection for a trait with a ${h}_v^2$ of 0.014 and ${c}_v^2$ of 0.012 (average values from Table 2) are equal to 0.234, 0.300, 0.364, and 0.412 for full-sib family sizes of 40, 80, 160, and 300, respectively. When the values of ${h}_v^2$ and ${c}_v^2$ reported by Janhunen et al. [10] are used, accuracies increase slightly and the required family sizes decrease. With the accuracies of sib selection above, it is possible to calculate expected changes in uniformity of BW. Using a proportion of 10% of selected animals (selection intensity = -1.755), a $C{V}_{a_v}$ of 0.211 in BE (Table 2), genetic gain was calculated following Mulder et al. [5]. Residual variance of BW (as a percent of the trait mean) decreased by -9%, -11%, -13%, and -15%, for full-sib family sizes of 40, 80, 160, and 300, respectively.

For many aquaculture species, it is possible to have large families. However, the challenge is that breeding candidates are from the offspring generation and their selection accuracy is lower than that of their parents. Progeny-testing schemes are effective to increase the accuracy of selection for traits with low heritability, including uniformity [5,28], but have not gained popularity in aquaculture breeding. In aquaculture breeding schemes, sib-testing is considered to be more feasible because large sib groups increase selection accuracy and generation intervals are shorter than in progeny-testing schemes. The most suitable designs to improve uniformity in aquaculture are still to be developed. Another approach to increase the accuracy of sib selection is to use genomic selection [37,38], which can theoretically increase accuracy of selection without progeny testing.

### Genotype by environment interaction

To the best of our knowledge, this is the first study on GxE interaction on uniformity of BW in rainbow trout. The main production environment for Finnish rainbow trout is in the Baltic Sea. PE and BE differ considerably in terms of water temperature, salinity, length of growing season, feeding practice, and type of cage culture (sea cages vs. earth-bottomed raceways). With standardized data, moderate re-ranking of families for uniformity of BW was found (*r*_g_ = 0.56), which indicates that uniformity of BW shares a certain degree of genetic background in each environment. When scale effects and micro-environmental sensitivity simultaneously influence uniformity of BW, the magnitude of re-ranking for uniformity of BW is only slightly smaller than for BW (0.62 to 0.70). This was surprising, given that the two environments analyzed differed greatly, and that standard deviations of individual BW, measured as squared residuals, are influenced by many unspecific abiotic and biotic environmental factors, as well as by developmental perturbations during the two years of growth. However, when the scale effect was accounted for by log-transformation, the genetic correlation between uniformity of BW in BE and PE decreased to -0.08. This shows that the scale effect may be the main factor that causes the moderate positive correlation between uniformity of BW in the two environments, and that uniformities estimated with standardized versus log-transformed data are genetically distinct traits. Log-transformed uniformity is more greatly influenced by micro-environmental sensitivity than by scale effects. The genetic correlation of -0.08 between uniformities of BW in BE and PE was the lowest genetic correlation reported for any trait across these two environments, but had a high standard error (0.33). The high sampling variance of the *r*_g_ may be explained by the low ${h}_v^2$ for uniformity of BW in both environments, but also by the fact that standard errors of genetic correlations tend to increase with decreasing magnitude of genetic correlations [39].

If the aim is to increase genetic response in uniformity of BW in both environments, an optimized selection strategy that accounts for the GxE interaction on uniformity and uses sib performances in both environments is recommended [40-42]. Uniformity of BW in the nucleus environment and in the production environment should be considered as two different traits and should be included separately in a selection index. In a situation where two or more environments are equally important, it might be possible to establish a separate breeding program for each environment to maximize response to selection for uniformity in each environment [43]. Nevertheless, establishing an additional breeding program is very costly and may not be possible in many cases.

### Genetic correlation between body weight and uniformity

In addition to the degree of re-ranking of families for uniformity in the two environments, the scale effect drastically affected the genetic correlation between BW and its uniformity. Genetic correlations of 0.30 and 0.79 were found between BW and its uniformity in BE and PE, respectively, but these values switched to -0.83 and -0.62 after the scale effect was accounted for by log-transformation. A similar change in sign was observed for correlations between BW in one environment and its uniformity in the other environment.

The data obtained for BE were also analyzed using the additive model, which used log-squared residuals [11]. This resulted in a genetic correlation of -0.40 between BW and its uniformity, which is closer to the genetic correlation estimated after log-transformation in the DHGLM. On the whole, the genetic correlation estimated between BW and its uniformity was unfavourable when untransformed BW data were used but favourable when log-transformed BW data were used. Hence, selection for BW increases the variance of BW, but decreases the coefficient of variation (*CV*) for BW because the increase in variance of BW is smaller than expected if the *CV* for BW remains constant or decreases. It may be argued that due to increased growth rate, farmers can harvest fish earlier rather than at an increased BW. Hence, the genetic gain in BW or growth (e.g. g/day) is expressed as lower age rather than higher weight at slaughter. In that case, it is still unknown whether genetic variation in uniformity of BW changes since fish are slaughtered at a younger age.

We observed a change in the magnitude and sign of genetic correlations between BW and its uniformity after data transformation, which agrees with other studies. Sonesson et al. [14] reported that the Pearson correlation between estimated breeding values of BW and its uniformity in Atlantic salmon changed from 0.42 with untransformed data to -0.17 with log-transformed data. Several studies on livestock species have reported negative genetic correlations between BW and its uniformity (${\overline{r}}_g$ = -0.36: min = -0.81 and max = -0.11) [6,11,22-24]. Yang et al. [29] estimated the genetic correlation between the mean and variance of litter size in rabbits and pigs using a model with Markov chain Monte Carlo (MCMC) sampling, which simultaneously estimated the model parameters and the Box-Cox transformation parameters. They compared genetic correlations before and after Box-Cox transformation and found that they changed from -0.73 to 0.28 for rabbit data and from -0.64 to 0.70 for pig data. Moreover, while the genetic trends across four successive generations showed that growth rate increased across generations by selection, no correlated genetic change in uniformity of growth rate was found during the same period, when uniformity was estimated based on log-transformed data (residual variation scaled by the trait mean). Estimates of genetic correlations between BW and its uniformity obtained in our study using the DHGLM and an additive model were of the same sign (-0.157) as in Janhunen et al. [10] but more negative. Thus, it is likely that selection for increased BW based on the genetic parameters estimated here, will result in a favourable correlated trend for log-transformed uniformity. Originally, it was suggested that mass selection for an increase in the mean of a trait may result in increased environmental variance because the selected extreme individuals may also have the highest micro-environmental sensitivity, even if there is no genetic correlation between the mean of the trait and its uniformity [4,5]. However, to test this hypothesis, it is necessary to consider whether the data on the trait is log-transformed or not. In other words, it is important to be explicit whether analysis of uniformity (or micro-environmental sensitivity) concerns the combined effect of scale and true micro-environmental sensitivity, or only the latter.

## Conclusions

We found a high potential for response to selection for uniformity of BW in rainbow trout relative to the mean and that uniformity of BW is a genetically different trait in breeding and production environments. We recommend that, in practice, aquaculture breeding programs use sib testing when the aim is to improve uniformity across environments. A large number of relatives will aid in obtaining a sufficiently high accuracy of selection for uniformity of BW. When using log-transformed harvest BW, we found a negative genetic correlation between BW and its uniformity, which indicates that selecting for increased BW and more uniform fish is possible. The scale effect substantially influences the genetic parameters for uniformity of BW, especially the sign and magnitude of the genetic correlations between BW and its uniformity and uniformity between environments.

## Appendix

The aim here is to show the extension of equation 16 in Mulder et al. [5] to multiple random effects and how other parameters such as *c*^2^ can be calculated. Felleki and Lundeheim [20] showed derivations to estimate the heritability of environmental variance. For completeness, they are provided here. In addition, we give equations for ${c}_v^2$, the proportion of variation in squared phenotypic deviations explained by common environmental effects.

First, the residual variance in the exponential model is calculated as:

1 $$ {\sigma}_{e, exp}^2={\sigma}_E^2/\left( \exp \left(\frac{1}{2}{\sigma}_{a_v, exp}^2\right) \exp \left(\frac{1}{2}{\sigma}_{c_v, exp}^2\right)\right), $$

where ${\sigma}_E^2$ is the residual variance from the mean model, assuming homogenous residual variance and assuming the use of an animal model.

Subsequently, as an analogy of equation 17 in Mulder et al. [5], the sum of the genetic variance and common environmental variance for the additive model can be calculated as:

2 $$ {\sigma}_{a_v}^2+{\sigma}_{c_v}^2={\sigma}_{e, exp}^4 \exp \left(2{\sigma}_{a_v, exp}^2\right) \exp \left(2{\sigma}_{c_v, exp}^2\right)-{\sigma}_E^4. $$

The product of equation (2) is a combination of ${\sigma}_{a_v}^2$ and ${\sigma}_{c_v}^2$. Under the assumption that the ratio of $\frac{\sigma_{a_v}^2}{\sigma_{a_v}^2+{\sigma}_{c_v}^2}$ is equal on both the additive and exponential scales, subsequently ${\sigma}_{a_v}^2$ is calculated as:

3 $$ {\sigma}_{a_v}^2=\left({\sigma}_{a_v}^2+{\sigma}_{c_v}^2\right)\frac{\sigma_{a_v, exp}^2}{\sigma_{a_v, exp}^2+{\sigma}_{c_v, exp}^2}, $$

and for common environmental effects:

4 $$ {\sigma}_{c_v}^2=\left({\sigma}_{a_v}^2+{\sigma}_{c_v}^2\right)\frac{\sigma_{c_v, exp}^2}{\sigma_{a_v, exp}^2+{\sigma}_{c_v, exp}^2}. $$

The heritability for environmental variance $\left({h}_v^2\right)$ can be calculated as:

5 $$ {h}_v^2=\frac{\sigma_{a_v}^2}{2{\sigma}_p^4+3\left({\sigma}_{a_v}^2+{\sigma}_{c_v}^2\right)}. $$

Similarly, the ratio between common environmental effects $\left({c}_v^2\right)$ can be calculated as:

6 $$ {c}_v^2=\frac{\sigma_{c_v}^2}{2{\sigma}_p^4+3\left({\sigma}_{a_v}^2+{\sigma}_{c_v}^2\right)}. $$

For these equations, it is assumed that genetic and common environmental correlations between mean and variance are 0; otherwise the denominator would be slightly higher with the exponential model (not shown). However, the effect of this simplifying assumption is negligible. The equations were verified with Monte Carlo simulation and following the same assumptions as in Mulder et al. [5].
